# Pharmacy practice research in Saudi Arabia; A bibliometrics analysis from (2000 to 2021)

**DOI:** 10.1016/j.jsps.2022.04.016

**Published:** 2022-04-30

**Authors:** Fawaz Mutlaq Alotaibi, Abdulaziz Abdullah Almotlak, Shakil Ahmad

**Affiliations:** a Pharmacy Practice Department, College of Clinical Pharmacy, Imam Abdulrahman Bin Faisal University, Dammam, Eastern Province, Kingdom of Saudi Arabia, P.O. Box 1982, Dammam 31441, Saudi Arabia; b Pharmacology Department, College of Clinical Pharmacy, Imam Abdulrahman Bin Faisal University, Dammam, Eastern Province, Kingdom of Saudi Arabia, P.O. Box 1982, Dammam 31441, Saudi Arabia; c Deanship of Library Affairs, Central Library, Building-A3, Imam Abdulrahman Bin Faisal University, Dammam, Eastern Province, Kingdom of Saudi Arabia, P.O. Box 1982, Dammam 31441, Saudi Arabia

**Keywords:** Bibliometric analysis, Pharmacy practice research, Saudi Arabia, Pharmacy research

## Abstract

**Background:**

Pharmacy is a growing profession in the Kingdom of Saudi Arabia which has experienced tremendous changes in the past 20 years. Pharmacy practice or clinical pharmacy have an attention ever since the emerging of PharmD programs throughout the Saudi universities. As a result, the number of affiliated faculty in the pharmacy practice departments has increased dramatically in the past 20 years and thus significant changes in research output were observed.

**Objectives:**

The main objective is to conduct a bibliometric analysis and evaluate the research output of pharmacy practice faculty in Saudi Universities from 2000 to 2021

**Methods:**

A systematic search was conducted using Scopus database to explore the research output from pharmacy practice affiliated faculty from 2000 to 2021. The following search terms AFFILORG (“Pharmacy Practice Department” OR “Department of Pharmacy practice” OR “Clinical Pharmacy Department” OR “Department of Clinical Pharmacy” OR “Department of Pharmacy Services”) AND AFFILCOUNTRY (“Saudi Arabia” OR “KSA” OR “Kingdom of Saudi Arabia”) were used. Only original research papers were retrieved and analyzed using MS Excel (v16.0), MS Access (v16.0), Bibexcel (v2017), VOS viewer, and Biblioshiny.

**Results:**

In the past two decades, most publications with pharmacy practice departments affiliation were pharmacy practice research irrelevant (only 1075 out of 2809). King Saud University and King Abdulaziz University were the top performing institutes, and median of 5-year impact factor for journals was more than 3 for most of the top 10 institutes. 19% of the total retrieved articles were review publications were the rest majorly classified as cross-sectional studies.

**Conclusion:**

The research contribution of pharmacy practice departments in Saudi Arabia has been improving. Key recommendations are to promote more applied and interventional research, increase publications in top journals, and enhance national collaborations.

## 1 Introduction:

Pharmacy, as a profession, went through several developmental phases in Saudi Arabia over the past few decades. This was evident by the massive expansion in the number of colleges of pharmacy around the Kingdom, which now exceeded 25 colleges that all are providing Doctor of Pharmacy or Bachelor of Pharmacy degrees (Alhamoudi, A., & Alnattah, 2018)(Badreldin et al., 2020a, Badreldin et al., 2020b). Part of the evolution of the pharmacy profession is the adaptation of clinical pharmacy, as an important discipline in pharmacy profession and integral part of high-quality healthcare system. The implementation of clinical pharmacy in the healthcare system has been widely applied in nearly all tertiary hospitals. These evolutionary phases would not have been possibly achieved without continuous investigation and research performed by the pharmacy practice experts. Pharmacy practice research is of a great importance in generating evidence that advance the field and maintain its vitality in the healthcare system. In fact, one of the main objectives of the national transformation program of the Saudi 2030 vision is to excel in providing the best healthcare services and improving the population quality of life (Chowdhury et al., 2021) (Ministry of Health, 2017). Therefore, it is very important to maintain the solid base of the profession and continuously generate scientific evidence that inform policymakers and strengthen the impact of the field to achieve the main goals of the country’s vision.

Pharmacy practice research is defined by the International Pharmaceutical Federation (FIP) as a type of research that emphasizes the impact of pharmacy practice on medication use, patient care and on the healthcare system (FIP, n.d.). In fact, the definition has grown to acquire other aspects of pharmacy profession that includes clinical, behavioral, economic, and humanistic impacts (FIP, n.d.). It has been shown previously that Saudi Arabia is leading the Arab world countries in term of the number of publications in the field of pharmacy practice (Sweileh, 2021). However, up to our knowledge, there is no published comprehensive review on the pharmacy practice research generated from Saudi’s faculties that assessed the performance of individual colleges and evaluated the quality of their publications. Here, we performed a bibliometric analysis on the contribution of all pharmacy practice departments from Saudi’s colleges of pharmacy published in the literature between January 2000 to September 2021. The main objective is to dissect the performance of all colleges and comprehensively provide a detailed overview that could aid the decision making and uplift the field.

## 2 Methods:

This is a descriptive study using a bibliometric analysis technique to analyze the pharmacy practice affiliated faculty publications from 2000 to Oct 2021 in Saudi Arabia. The retrieved studies were from Scopus, a very broad databases that included majority of research article in the field. A systematic search was conducted using the following search terms, AFFILORG (“Pharmacy Practice Department” OR “Department of Pharmacy practice” OR “Clinical Pharmacy Department” OR “Department of Clinical Pharmacy” OR “Department of Pharmacy Services”) AND AFFILCOUNTRY (“Saudi Arabia” OR “KSA” OR “Kingdom of Saudi Arabia”). This means, any article that was published by a pharmacy practice affiliated faculty in Saudi Arabia from 2000 to 2021 was retrieved. Although the name of pharmacy practice department is not consistent with all pharmacy colleges in Saudi Arabia, a search has been conducted by the authors to sort all Saudi university that has college of pharmacy/college of clinical pharmacy and a list of pharmacy practice department names was created and therefore used in the search engine.

### 2.1 Inclusion and exclusion Criteria:

The search was limited to research articles as of our study objectives. The following filters have been applied, which include language, geographical and date filters. All type of study design were included in the study, such as randomized control trials, cross sectional studies, case control studies, and retrospective cohort studies. In addition, Systematic review, literature review, book chapters, and letter to the editor have been excluded in the analysis.

### 2.2 Data extractions:

The data was extracted from the search engine had the following information: study title, author names, author affiliations, keywords, title of the study, abstract, year of publication, journal impact factor, H-index of the journal and study type whether original research paper or review paper. After applying and inclusion and exclusion criteria, and based on the main objective of the study and to eliminate selection bias, two independent reviewers carried out the search process. Any conflicts in the retrieved articles, a third reviewer resolve the conflict. The independent reviewers followed the following steps to meet the study objectives after ensuring that the study has a pharmacy practice affiliated faculty; whether the study is pharmacy practice related. This classification is based on the definition of International Pharmaceutical Federation for pharmacy practice research (FIP, n.d.). Further classification was applied according to the study design, including randomized control trials, cross-sectional studies, retrospective cohort studies, case control studies, and case report/case series, which represent most of the study designs were seen by the reviewers during the search process. These data as of October 2021.

**Fig. 1:**
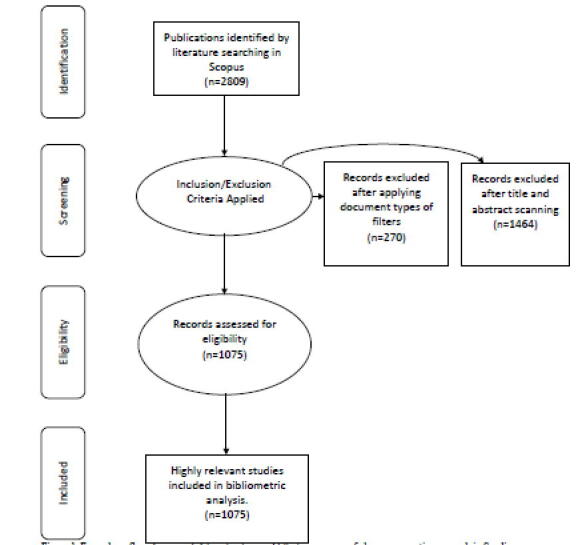
Four-phase flow diagram of data extraction and filtration process of pharmacy practice research in Saudi Arabia.

The initial result consisted of 2809 documents, and after screening all documents, 1734 were found irrelevant records that represent fields other than pharmacy practice and were removed from analysis. The remaining 1075 articles were exported into MS Excel Format. The accuracy of the data was ensured by repeating the process by two team members of the research group. **(**Fig. 1**)**.

#### 2.2.1 Data Analysis:

The analyses and visualization of the 1075 selected articles and their related data were performed using various tools and software including MS Excel (v16.0), MS Access (v16.0), Bibexcel (v2017) free software for bibliometric analysis, VOS viewer (version 1.6.17, Leiden University, The Netherlands), Biblioshiny (version 3.1.4, University of Naples Federico II, Italy) (Aria and Cuccurullo, 2017, Persson et al., 2009, Van Eck and Waltman, 2010).

## 3 Results:

### 3.1 Overall pharmacy practice research output:

**Fig. 2:**
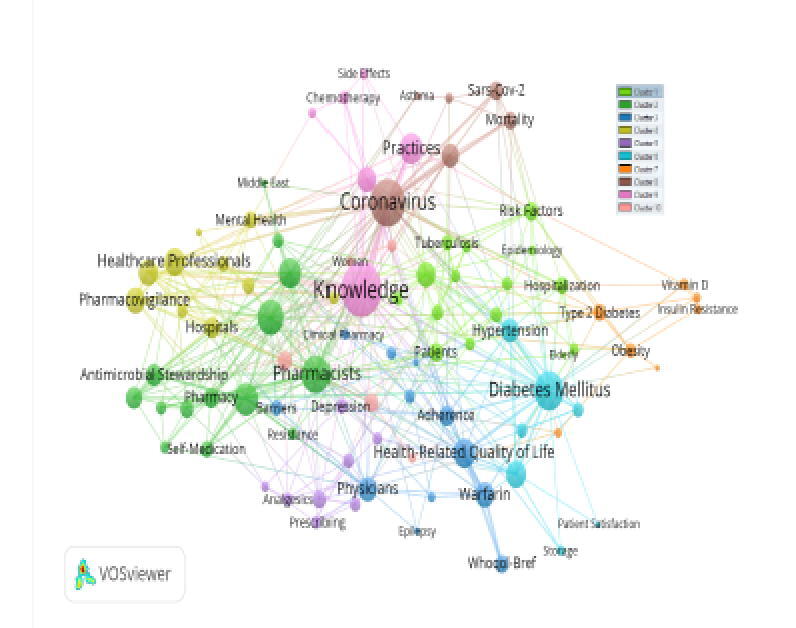
Keywords evolution of pharmacy practice research over 2000–2021.

From 2000 till October 2021, and after applying research query, we have identified 1075 out of 2809 articles that represent pharmacy practice field of research. As shown in Table 1 this is nearly 40% of the total retrieved articles with pharmacy practice affiliations in Saudi Arabia. Out of the 1075 articles, 17% of them were review articles (184 out of 1075) and 92% of the publications were cross-sectional based studies (992 out of 1075). The remaining 83 articles were either clinical trials or case series and case report studies. Over the years, there has been a steady increase in the number of research publications in the field of pharmacy practice. As shown in Table 2, the number of publications grew from only one publication in 2000 to 268 publications in 2021. Data for citations numbers, average citations per publication, and other metrics were also shown in Table 2. The 1075 identified articles represent a wide range of research domains that include social pharmacy, medication adherence, antibacterial stewardship, clinical pharmacogenomics, Pharmacoeconomics and health-related quality of life, and many other fields (Fig. 2).

**Table 1:** Main information about data of pharmacy practice research.

**Description**	**Results**
Periods	Jan 2020:Oct 2021
Sources (Journals, Books, etc.)	407
Documents	1075
Cited Documents	727
Non-Cited Documents	348
Review articles	184
Total Authors	3595
Single-authors	44
Multi-authors	3551
Total citations	6512
Average citations per documents	6.058
Original research	891
Cross-sectional studies	992
Author's keywords	2546

**Table 2:** Citation structure of pharmacy practice research.

**Year**	**TP**	**TCP**	**CTP**	**TC**	**C/P**	**C/CP**	**C/CTP**	**h-index**
2000	1	1	1	2	2.00	2.00	2.00	1
2001	4	3	5	10	2.50	3.33	2.00	1
2003	4	3	9	128	32.00	42.67	14.22	3
2004	4	4	13	52	13.00	13.00	4.00	3
2005	3	2	16	10	3.33	5.00	0.63	2
2006	3	3	19	65	21.67	21.67	3.42	2
2007	5	5	24	56	11.20	11.20	2.33	4
2008	8	6	32	67	8.38	11.17	2.09	5
2009	5	5	37	74	14.80	14.80	2.00	4
2010	11	11	48	223	20.27	20.27	4.65	8
2011	24	22	72	468	19.50	21.27	6.50	10
2012	41	41	113	823	20.07	20.07	7.28	16
2013	32	30	145	574	17.94	19.13	3.96	15
2014	45	43	190	695	15.44	16.16	3.66	15
2015	45	37	235	463	10.29	12.51	1.97	12
2016	32	29	267	315	9.84	10.86	1.18	10
2017	75	70	342	515	6.87	7.36	1.51	11
2018	103	86	445	653	6.34	7.59	1.47	14
2019	130	101	575	623	4.79	6.17	1.08	11
2020	232	152	807	537	2.31	3.53	0.67	10
2021	268	73	1075	159	0.59	2.18	0.15	6

TP (total publications), TC (total citations), TCP (total of cited publications), CTP (cumulative total publications), C/P (average citations per publication), C/CTP (average citations per cumulative publication), C/CP (average citations per cited publication).

### 3.2 Colleges of pharmacy contribution:

Looking at the contributions of Colleges of Pharmacy in the Kingdome and as expected, we identified that nearly 40% of the 1075 documents were published by researchers in King Saud University (KSU) followed by King Abdulaziz University (KAU) (10%). Other top ten were shown in Table 3 in which Less than 90 publications were identified from Prince Sattam bin Abdulaziz University (PSAU), King Khaled University (KKU), Qassim University (QU), Imam Abdulrahman Bin Faisal University (IAU), Princess Nourah Bint Abdulrahman University (PNU), King Saud Bin Abdulaziz University for Health Sciences (KSAU-HS), Taif University (TU) and King Faisal University (KFU).

**Table 3:** Leading Organizations in publications on pharmacy practice research.

**Rank**	**Affiliation**	**TP**	**TCP**	**TC**	**C/P**	**C/CP**	**IF-2020**	**5 Year IF**
1	King Saud University	434	336	3703	8.53	11.02	3.336	3.788
2	King Abdulaziz University	102	73	777	7.62	10.64	3.411	3.745
3	Prince Sattam Bin Abdulaziz University	87	38	120	1.40	3.16	2.463	2.703
4	King Khalid University	85	55	349	4.11	6.35	3.200	3.777
5	Qassim University	84	58	433	5.15	7.47	2.711	3.117
6	Imam Abdulrahman Bin Faisal University	72	50	338	4.69	6.76	3.200	3.297
7	Princess Nourah Bint Abdulrahman University	68	32	205	3.06	6.41	3.220	3.618
8	King Saud Bin Abdulaziz University for Health Sciences	60	42	197	3.28	4.69	2.487	2.724
9	Taif University	47	31	216	4.60	6.97	2.946	2.885
10	King Faisal University	39	21	140	3.59	6.67	3.048	3.341

Table shows TP (total publications), TC (total citations), TCP (total cited publications), C/P (average citations per publication), C/CP (average citations per cited publication), IF (impact factor), of top 10 leading organizations in Saudi Arabia.

### 3.3 Quality of pharmacy practice research as determined by impact factor and journal quartile:

To assess the quality of publications, we sought to determine the impact factors for the journals where the top performing universities tend to publish their articles. The median 5-year impact factor for all publications from each university was determined. As shown in Table 3, 7 out of 10 evaluated universities had a median 5-year impact factor of more than 3, while three universities (PSAU, KSAU-HS) and TU showed a median 5-year impact factor of less than 2. Number of citations per publication was also determined, as another determinator of research impactfulnees. The average number of citations varies between universities, and the top two performing universities (KSU and KAU) have an average citations number of more than 7, while others mostly have average 5 or less citations per publication.

Further assessment of the publications, we determined the top 20 sources of publications that Pharmacy practice researchers tend to publish their articles. Speaking of which, and as shown in Table 4, these 20 journals are rarely classified in the first quartile, the top 25% journals in the field. As expected, the top publishing journal was the Saudi Pharmaceutical Journal, a journal affiliated to King Saud University with a total number of publications of 102 (nearly 10% of the total pharmacy practice analyzed data). Only 3 journals out of 20 were classified as Q1 journals, representing less than 15% of the published articles by the top 20 journals (276 out of 1075). Surprisingly, some of the journals are classified as Q4 with unknown impact factor. These were seen in 53 articles, which represents 20% of all published articles among the top 20 journals (Table 4).

**Table 4:** Most Preferred journals publishing pharmacy practice research of affiliated authors.

**Journal**	**NP**	**NC**	**C/P**	**Country**	**Publisher**	**IF-2020**	**Q-2020**	**5 Y-IF**	**h_index**
Saudi Pharmaceutical Journal	105	1008	9.60	Saudi Arabia	Elsevier	4.330	2	4.895	18
Plos One	19	276	14.53	USA	Public Library Science	3.240	1	3.788	8
Latin American Journal of Pharmacy	14	27	1.93	Argentina	Colegio Farmaceuticos Provincia De Buenos Aires	0.249	4	0.250	3
Saudi Medical Journal	14	151	10.79	Saudi Arabia	Saudi Med J	1.484	3	2.051	7
Tropical Journal of Pharmaceutical Research	13	38	2.92	Nigeria	Pharmacotherapy Group	0.533	4	0.743	4
International Journal of Clinical Pharmacy	12	69	5.75	Netherlands	Springer	2.054	3	2.474	5
International Journal of Environmental Research and Public Health	11	24	2.18	Switzerland	MDPI	3.390	1	3.789	2
Risk Management and Healthcare Policy	11	31	2.82	New Zealand	Dove Medical Press Ltd	3.200	2	4.527	3
Annals of Saudi Medicine	9	70	7.78	Saudi Arabia	K Faisal Spec Hosp Res Centre	1.526	3	2.044	4
Journal of Infection and Public Health	9	86	9.56	England	Elsevier	3.718	2	3.73	6
Antimicrobial Agents and Chemotherapy	8	54	6.75	USA	Amer Soc Microbiology	5.191	1	5.346	5
Journal of Pharmacy and Bioallied Sciences	8	65	8.13	India	Wolters Kluwer Medknow	N/A	4	N/A	4
Research in Social and Administrative Pharmacy	7	90	12.86	USA	Elsevier	3.336	2	3.532	6
Antibiotics-Basel	6	23	3.83	Switzerland	MDPI	4.639	2	4.849	3
BMC Health Services Research	6	78	13.00	England	BMC	2.655	3	3.297	4
BMJ Open	6	60	10.00	England	BMJ	2.692	2	3.424	4
Journal of Applied Pharmaceutical Science	6	17	2.83	India	Medipoeia	N/A	N/A	N/A	3
Journal of Clinical and Diagnostic Research	6	53	8.83	India	Premchand Shantidevi Research Foundation	N/A	3	N/A	4
Journal of Taibah University Medical Sciences	6	35	5.83	Saudi Arabia	Elsevier	N/A	3	N/A	5
Patient Preference and Adherence	6	70	11.67	England	Dove Medical Press Ltd	2.711	2	3.117	5

Table displays NP (number of publications), NC (number of citations), IF (Impact Factor of source), Q (Quartile), publisher and county of 20 preferred journals in global information retrieval research.

### 3.4 Extent of international collaboration:

**Fig. 3:**
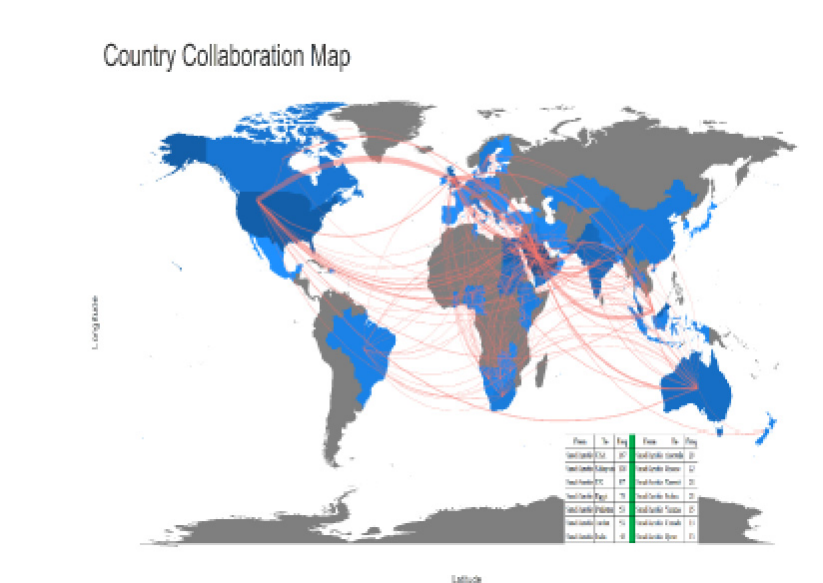
Top collaborative countries of pharmacy practice affiliated faculty.

In terms of international collaboration, many countries from different continents have been identified. As shown in Table 5 **and** Fig. 3, pharmacy practice researchers in Saudi Arabia tend to collaborate with mostly researchers from United States, Malaysia and UK based institutes. USA is the top more than 180 articles were coauthored by one or more collaborators from USA. Malaysia comes as the second with 109 articles and UK is the third with 87 articles. The average number of citations per publication significantly varies between those collaborative countries, with some collaborative countries exceeding 30 citations and others less than 3 citations per publication.

**Table 5:** Leading collaborative countries in publications on pharmacy practice research.

**Country**	**TP**	**TC**	**C/P**	**h-index**
USA	186	956	5.14	16
Malaysia	109	643	5.90	14
UK	87	932	10.71	17
Egypt	77	410	5.32	11
Pakistan	53	245	4.62	9
Jordan	52	129	2.48	6
India	40	161	4.03	8
UAE	31	121	3.90	6
Australia	29	295	10.17	8
Greece	22	826	37.55	15
Sudan	20	184	9.20	9
Kuwait	20	60	3.00	5
Yemen	15	57	3.80	5
Qatar	13	39	3.00	4
Canada	13	119	9.15	3
Italy	10	207	20.70	7
Sweden	8	197	24.63	7
South Africa	8	79	9.88	6
Lebanon	8	42	5.25	3
Hong Kong	6	82	13.67	3

Table shows TP (total publications), TC (total citations), C/P (average citations per publication), and h-index of top 20 leading countries in global information retrieval research.

## 4 Discussion:

To the best of our knowledge, this is the first bibliometric analysis to investigate the pharmacy practice research output in the past two decades in the Kingdome of Saudi Arabia.

An expected increase in the pharmacy practice research over the past 20 years in KSA has been due to the expansion of establishing more than 20 colleges of pharmacy since 2003 as a result of establishing of more than 28 government or private universities (Aljadhey et al., 2017). Moreover, King Abdullah scholarship program was established during the same time where many students were awarded a fully funded scholarship to pursue their undergraduate or postgraduate education in United Kingdom or north America (Alqahtani, 2014). Such increase, enrich the literature in different fields in the pharmacy in general and especially in the pharmacy practice. Another supporting evidence for the tremendous increase has been due to the increased attention/publicity of the PharmD/residency programs in the past 10 years, which attracts pharmacy graduates to pursue their post-graduate degrees in clinical practice (Korayem et al., 2021). This increase can be also attributed to the requirements of residency programs offered by the Saudi Commission for Health Specialties that research record is one of the criteria for evaluation and acceptance (Specialities, 2021).

Although 40% of the total affiliated pharmacy practice research is focusing on pharmacy practice type of questions, the remaining research output is in different sub-specialties such as pharmacology, medicinal chemistry, and natural products. However, there are many justifications for pharmacy practice researchers to embark on research projects in fields other than pharmacy practice. These include participating in writing the manuscript, generating the research questions or ideas, or proofreading the manuscript to their colleagues in other departments (Guidelines, 1993), (Minshew and McLaughlin, 2019), (International Committee of Medical Journal Editors, 2021). Another interesting finding was that around 90% of the retrieved publications were cross-sectional types of studies and the vast majority of them were survey-based research. It is obvious that absence of national medical data, difficulty in contacting government or non-government hospitals, lack of interest in some faculty to do a study based on real-world data, lack of research assistants to help in data collection and data analysis, shortage in clinical graduate programs, which limit the number of Master and Ph.D. students, the increase in the administrative and teaching load on the faculty are reasons to be involved in such research (International Committee of Medical Journal Editors, 2021), (Sweileh, 2021). In contrast, KSU has post-graduate programs, medication safety research chair, which clearly reflects that in our findings by ranking the top university in terms of research activity (University, 2022). The participation of pharmacy practice faculty in clinical trials conducted in the listed institutions is extremely low despite the high impact and potential of these studies to be conducted in the Kingdom. To overcome this issue, we suggest establishing a consortium or annual meetings for all pharmacy practice researchers and all faculty who is interested in clinical research to meet and exchange research ideas, proposed solutions for young researchers, train young leaders in the field of pharmacy practice, and creating a program of mentorship for the new researchers or graduate students nationally and internationally. This initiative could be led by the Saudi Society of Clinical Pharmacy, an optimistic association established in 2021 (Pharmacy, 2022).

The study findings show that King Saudi University (KSU) and King Abdulaziz University (KAU) are the top two institutions in terms of Pharmacy practice research contributions in the past 20 years. This is, in fact, due to the seniority of the two institutions. The college of pharmacy in the two institutions was established in 1959 and 2001, respectively. Although 40% of the research contributions from KSU, the publications were published in journals with comparable 5-years median impact factors (IF = 3.78) to other young institutions such as King Khalid University (IF = 3.77), Princess Nourah Bint Abdulrahman University (3.61), and Imam Abdulrahman Bin Faisal University (3.29). In addition, the average citation per publication is quite similar between KSU (C/P = 8.53) and KAU (C/P = 7.62) despite the enormous difference in the total number of publications, 434 and 102, respectively.

It is worth mentioning that some of the top 10 universities, such as KSU, KAU, IAU, KSAU-HS and PNU (shown in Table 4), have affiliated hospitals, research chairs and well-known research centers that provide good research opportunities for their affiliated researchers. However, in some of the previously mentioned ones, the research output in pharmacy practice is not hugely different than those who lack such resources, either in the total output of the quality of research. In fact, having an affiliated hospital to the university is a major advantage for the faculty of pharmacy that we think is not yet being efficiently utilized.

The international collaborations were observed more in the USA, Malaysia, and UK as previous studies have reported (Sweileh, 2021). This observation is because many of the pharmacy practice scholars in these universities have completed their post-graduate degrees in these countries and tend to build their research network and continue to do so after graduation (Badreldin et al., 2020a, Badreldin et al., 2020b). A large number of schools of pharmacy and high-quality research are another explanation of such collaboration with US and UK universities (Sweileh, 2021). For the same reason, the collaboration between Saudi institutes researchers with USA or UK-based authors is observed across many other disciplines.

Although universities have implemented incentive programs for researchers publishing in high quality journal, our findings show that some of the retrieved articles were published in Q3 or Q4. This could be explained by those publications being published either for a personal motive or low quality of data that will not get the chance to be published in decent quality journals. Another explanation could be that most of the publications have been performed on local population, thus the result might not be generalizable, therefore might not be accepted in high quality international journals.

### 4.1 Limitations:

This study has several limitations. First, we heavily relied only on the affiliation of the researchers to retrieve the publications, which could underestimate the number of publications of pharmacy practice faculty. Second, although we have used only Scopus database, which is one of the largest database there is a chance that we could miss other published work in other databases (Aksnes and Sivertsen, 2019, Kiduk and Meho, 2006). Third, we focused only on the research institutions, which means we did not focus on the publications that came from hospital-based affiliations as per the research interest. Last, we looked at the international collaborations with the Saudi Universities, but we were not able to look at the national level of collaborations due to the complexity of the database to answer such question.

### 4.2 Suggestions for future research:

There are a couple of research questions we have generated after finishing this study. One question could test the level of a national collaboration between the Saudi college of pharmacy. Another question is related to the common them that most Saudi colleges of pharmacy conduct their research about. Lastly, the level of involvement of hospital pharmacy in research related to pharmacy practice specifically or clinical trials.

## 5 Conclusion:

The study aims to analyze the pharmacy practice research output conducted by Saudi Universities in the past two decades using the bibliometric analysis approach. The study finds that only 40% of pharmacy practice department research was related to pharmacy practice, and KSU and KAU were the top two publishing institutions. Pharmacy practice researchers and respected stakeholders are encouraged to meet in an annual meeting to discuss the opportunity for collaborations, brainstorm future research questions, create programs to support new researchers, and mentorship them.

## Declaration of Competing Interest

The authors declare that they have no known competing financial interests or personal relationships that could have appeared to influence the work reported in this paper.

## References

[b0005] Aksnes D.W., Sivertsen G. (2019). A criteria-based assessment of the coverage of scopus and web of science. J. Data Inf. Sci..

[b0010] Alhamoudi A., Alnattah A. (2018). Pharmacy education in Saudi Arabia: the past, the present, and the future. Curr. Pharm. Teach. Learn..

[b0015] Aljadhey H., Asiri Y., Albogami Y., Spratto G., Alshehri M. (2017). Pharmacy education in Saudi Arabia: A vision of the future. Saudi Pharm. J..

[b0020] Alqahtani A. (2014). Evaluation of King Abdullah Scholarship Program. J. Educ. Pract..

[b9000] Aria M., Cuccurullo C. (2017). bibliometrix: An R-tool for comprehensive science mapping analysis. Journal of informetrics.

[b0025] Badreldin H.A., Alosaimy S., Al-jedai A. (2020). c. ournal Am. Coll. Clin. Pharm..

[b0030] Badreldin H.A., Alosaimy S., Al-jedai A. (2020). Clinical pharmacy practice in Saudi Arabia: Historical evolution and future perspective. JACCP J. Am. Coll. Clin. Pharm..

[b0035] Chowdhury S., Mok D., Leenen L. (2021). Transformation of health care and the new model of care in Saudi Arabia: Kingdom’s Vision 2030. J. Med. Life.

[b0040] FIP, n.d. Pharmacy practice research—FIP—International Pharmaceutical Federation. [WWW Document]. URL https://www.fip.org/pharmacy-practice-research.

[b0045] Guidelines A. (1993). Authorship guidelines agreed. Nurs. Stand..

[b0050] International Committee of Medical Journal Editors, 2021. Recommendations for conduct, reporting, editing, and publication of scholarly work inmedical journals. 1–19. 10.12771/emj.2024.e48 PMC12093628 40704003

[b0055] Kiduk, Y., Meho, L.I., 2006. Citation analysis: A comparison of google scholar, scopus, and web of science. Proc. ASIST Annu. Meet. 43. https://doi.org/10.1002/meet.14504301185

[b0060] Korayem G.B., Badreldin H.A., Eljaaly K., Aldemerdash A., Al-Suhaibani L.K., Joharji H., Aljuhani O., Al-Omari B.A., Almudaiheem H.Y., Alhifany A.A., Alawagi M., Al-Mowaina S.M., Al-Jazairi A.S., Albekairy A.M., Al-Jedai A. (2021). Clinical pharmacy definition, required education, training and practice in Saudi Arabia: A position statement by the Saudi society of clinical pharmacy. Saudi Pharm. J..

[b0065] Ministry of Health (2017). Health Sector Transformation Strategy. Minist. Heal..

[b0070] Minshew L.M., McLaughlin J.E. (2019). Authorship considerations for publishing in pharmacy education journals. Am. J. Pharm. Educ..

[b0075] Pharmacy, S.S. of C., 2022. Saudi Society of Clinical Pharmacy [WWW Document].

[b9005] Persson, O., Danell, R., Schneider, J. W. (2009). How to use Bibexcel for various types of bibliometric analysis. Celebrating scholarly communication studies: A Festschrift for Olle Persson at his 60th Birthday, 5, 9–24.

[b0080] Specialities, S.C. for health, 2021. Saudi Commission for Health Specialties Matching System Principles and Guidelines 2020–2021.

[b0085] Sweileh W.M. (2021). Contribution of faculties of pharmacy in Arab countries to pharmacy practice research: a bibliometric analysis (1990–2020). Int. J. Pharm. Pract..

[b0090] University, K.S., 2022. Deanship of Graduate studies, King Saud University [WWW Document].

[b9010] Van Eck N., Waltman L. (2010). Software survey: VOSviewer, a computer program for bibliometric mapping. Scientometrics.

